# Supplementary figures and images for: Ecological landscapes guide the assembly of optimal microbial communities
Source: PLoS Comput Biol. 2023 Jan 10;19(1):e1010570. doi: 10.1371/journal.pcbi.1010570 (PMC9831326; doi:10.1371/journal.pcbi.1010570)

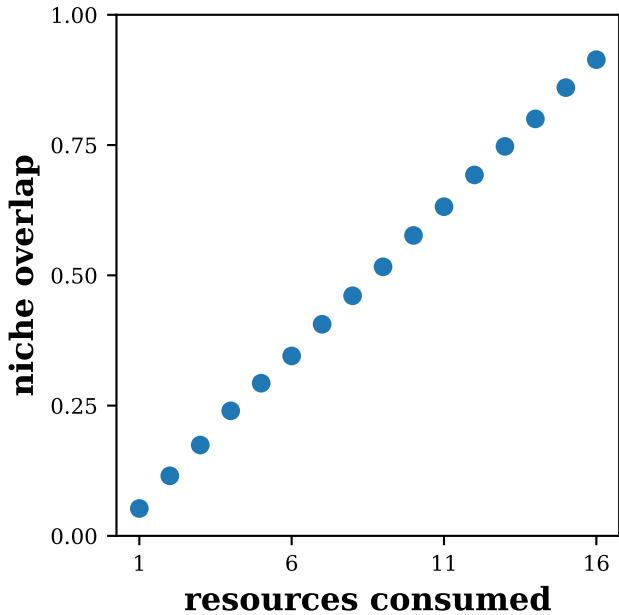

Supplement: S1 Fig — The niche overlap in the 16 species pools shown in Fig 2. Niche overlap was quantified by the average cosine similarity between the consumption vectors of each species pair in the pool, as in previous studies [33]. In these simulations, each non-zero consumption matrix element was drawn from a gamma distribution. The niche overlap increased with number of resources when consumption matrix elements were drawn from other distributions as well. See S11 Fig. (PDF) [file pcbi.1010570.s002.pdf]

**A**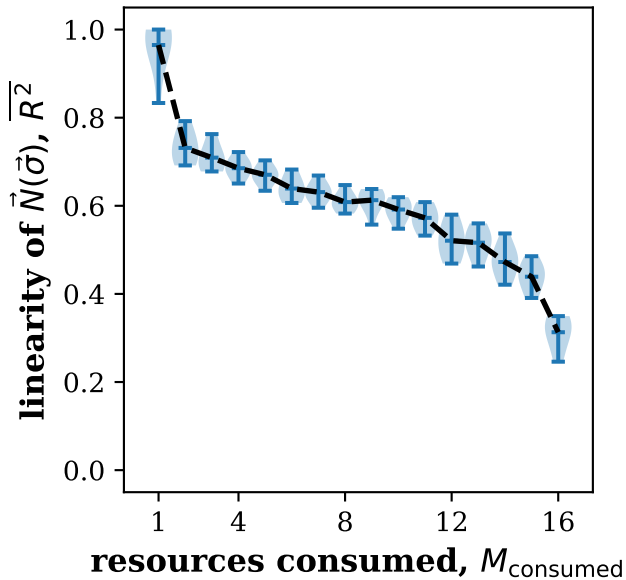**B**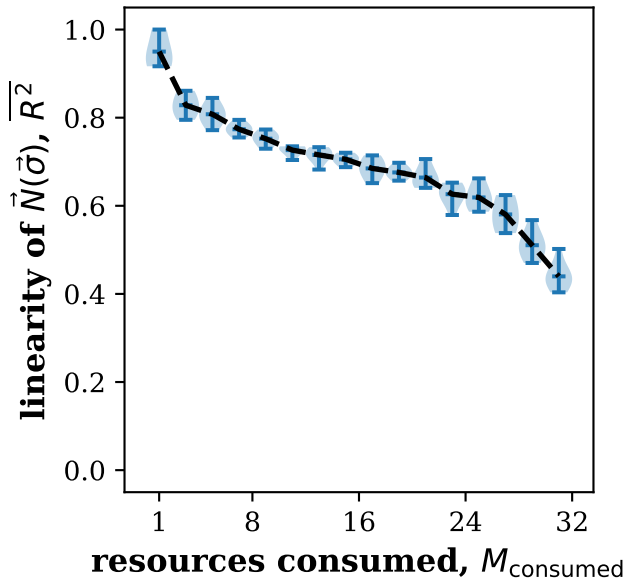**C**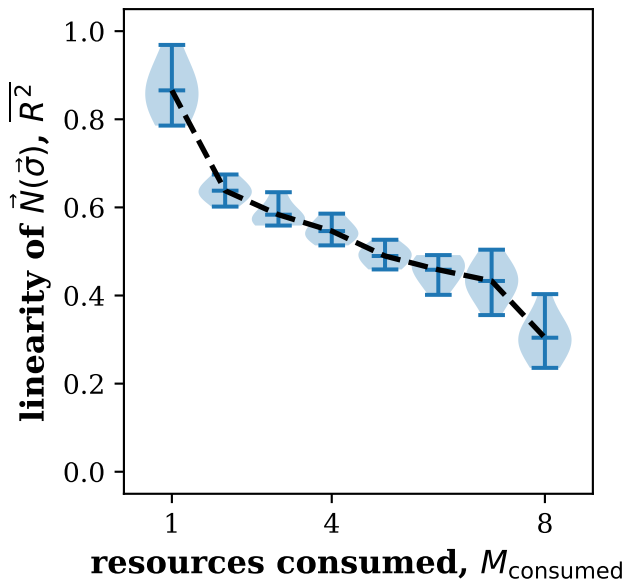**D**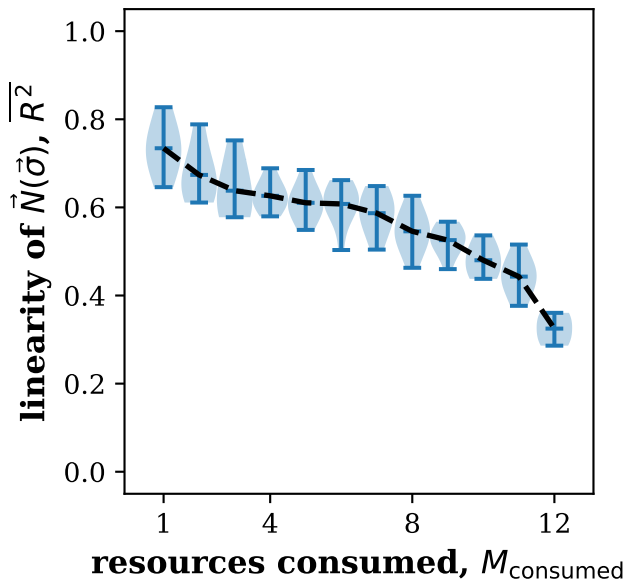

Supplement: S2 Fig — The map from species presence to steady-state abundances N→(σ→) becomes more nonlinear with increasing niche overlap across a range of models and parameters. The linearity of the map is quantified by R2¯. Niche overlap increases when species in the pool consume more resources. We plot consumer resource models with S = 16, Mtot = 16 in (A), S = 16, Mtot = 32 in (B), and S = 16, Mtot = 8 in (C); and cross-feeding model with S = 12, Mtot = 12 in (D). There were 10 replicates for each data point. Simulation parameters are described in Methods. (PDF) [file pcbi.1010570.s003.pdf]

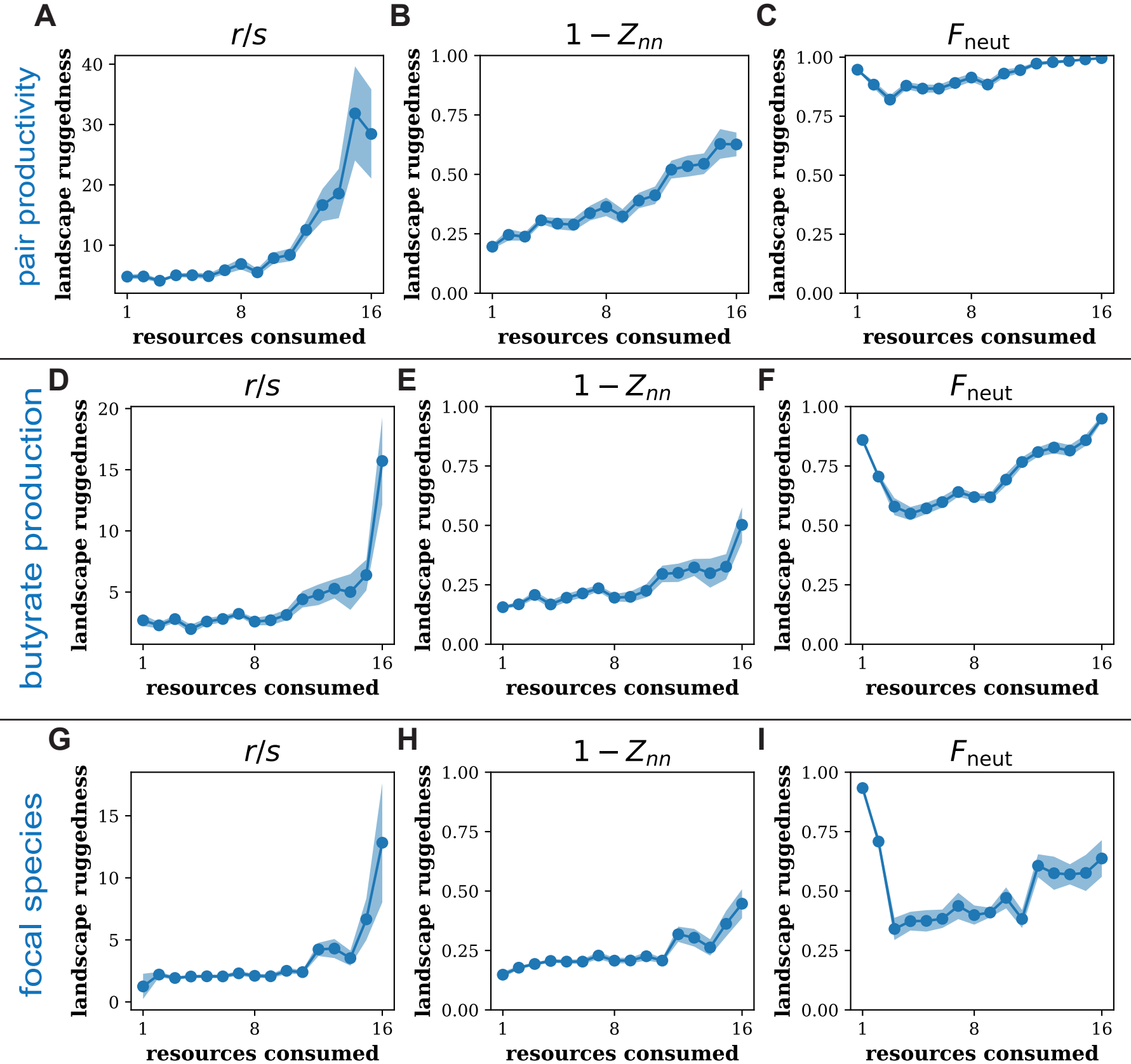

Supplement: S3 Fig — Panels show r/s, 1 − Znn, and Fneut the landscapes with community function being pair productivity, butyrate production, and focal species abundance. The fraction of neutral directions Fneut was high at low niche overlap, when all of the species occupied separate niches, because the species (or species pair) responsible for the community function was unaffected by the addition or removal of other species. This causes the community function to be left unchanged and a concordant increase in the number of neutral directions. Simulations were the same as in Fig 5. (PDF) [file pcbi.1010570.s004.pdf]

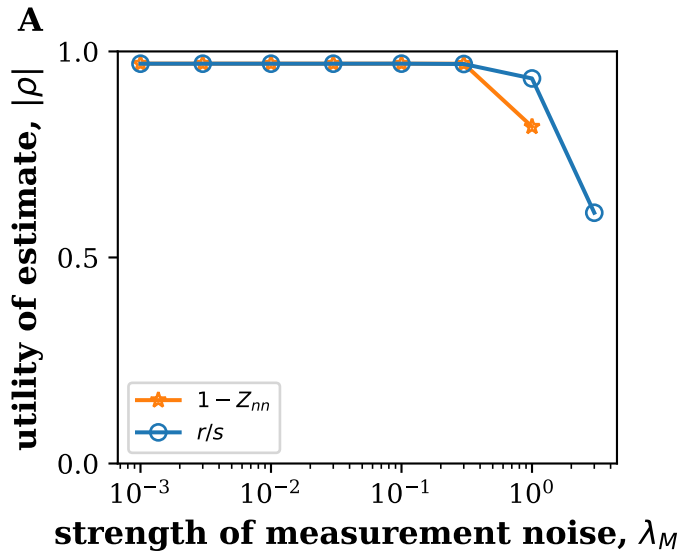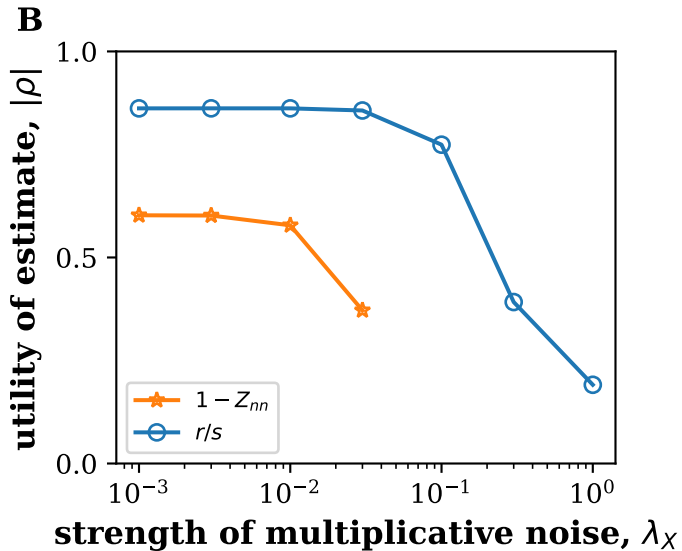

Supplement: S4 Fig — Magnitude of the correlation between ruggedness estimated from noisy data and search efficacy remains high even when experimental noise is as large as the community function itself. Two forms of noise were simulated, additive noise in measurement (A) and multiplicative noise (B). The strength of the noise λM and λX is measured relative to the community function; therefore a noise strength of one means that the contribution from noise is as large as the community function itself. The community function was the productivity of a pair of species in panel A and diversity in panel B. Noise was simulated as described in SI. Data was obtained from simulations in Fig 5. (PDF) [file pcbi.1010570.s005.pdf]

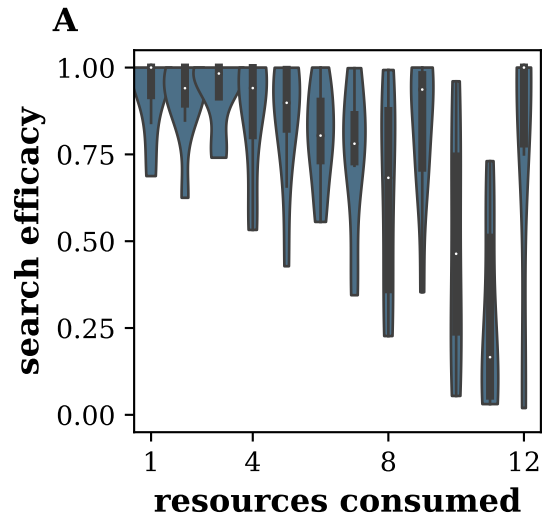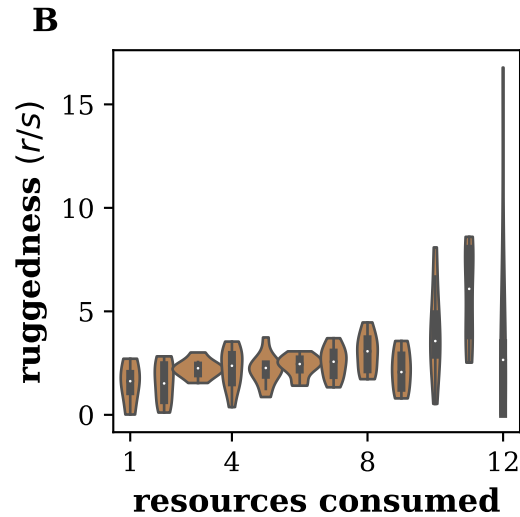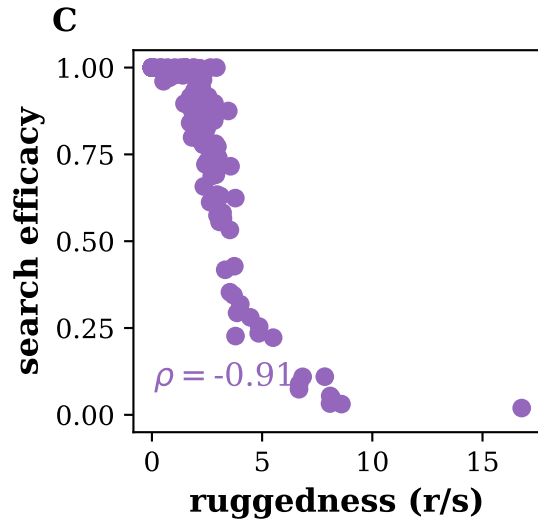

Supplement: S5 Fig — (A,B) Search outcome and ruggedness of a cross-feeding model where the niche overlap between species was varied by changing the number of resources consumed by each species, mirroring results obtained in model without cross-feeding (Figs 3 and 5). (C) Ruggedness remained informative of search efficacy. Community function was the abundance of a focal species. Parameters are described in Methods. (PDF) [file pcbi.1010570.s006.pdf]

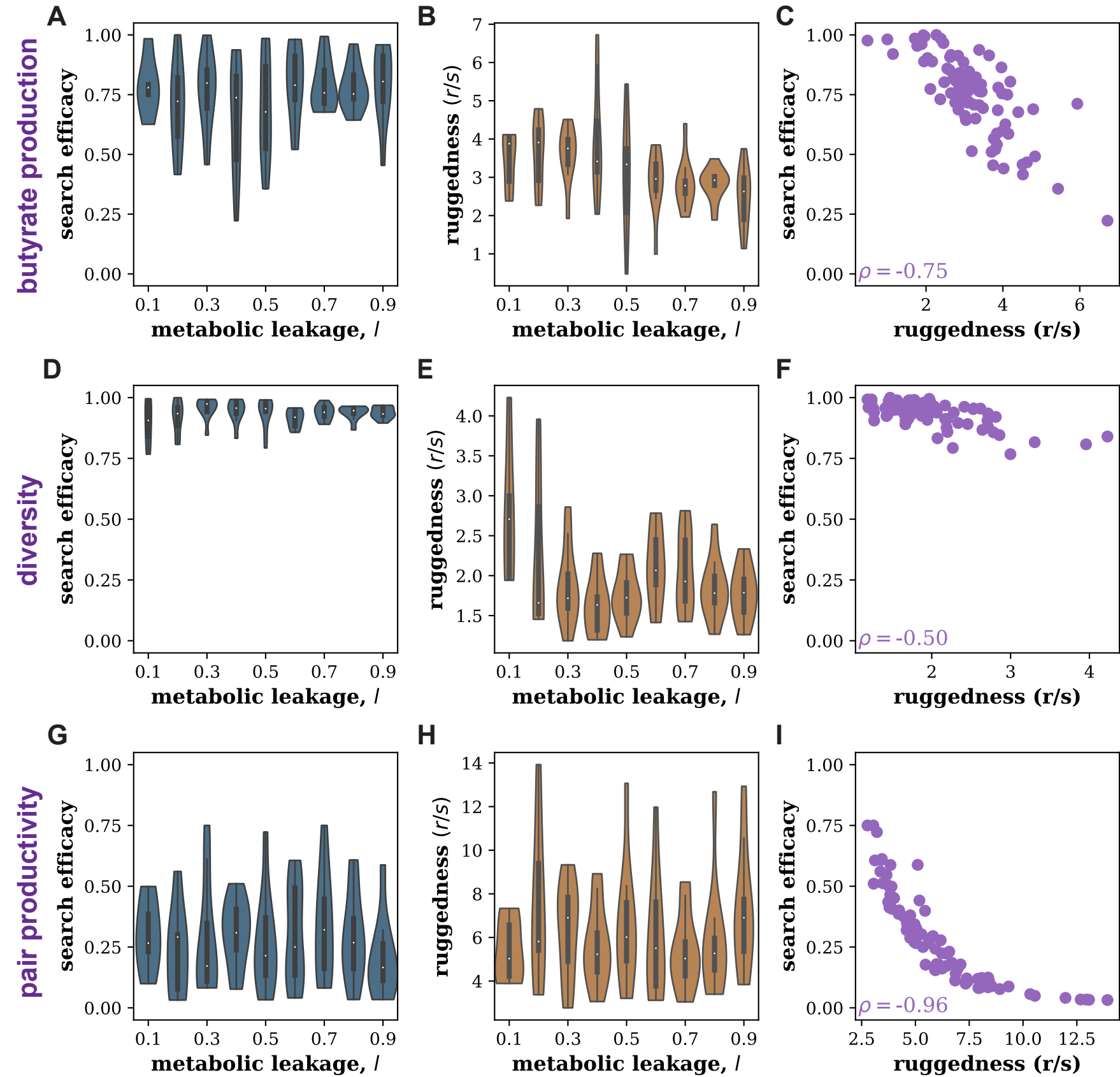

Supplement: S6 Fig — Panels demonstrate the search efficacy, ruggedness, and correlation between search efficacy and ruggedness on landscapes with community functions of butyrate production, diversity, and pair productivity. All reported correlations were statistically significant. (PDF) [file pcbi.1010570.s007.pdf]

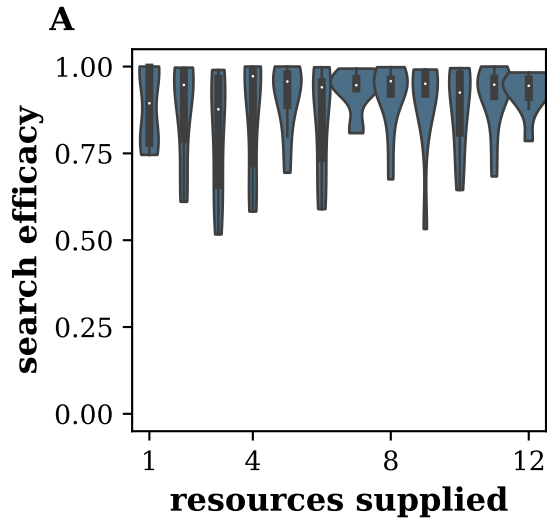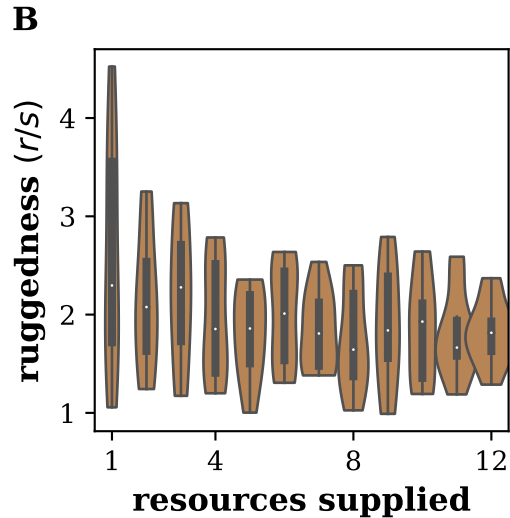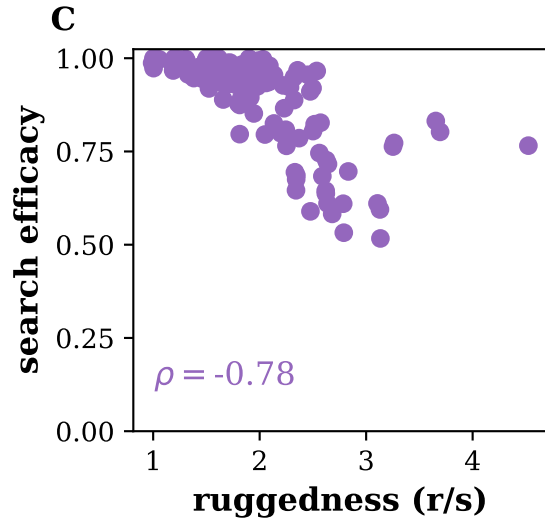

Supplement: S7 Fig — (A,B) Search efficacy and ruggedness of a cross-feeding model where the number of resources supplied to the community was varied. (C) Ruggedness remained informative of search efficacy. Community function was the abundance of a focal species. The total amount of resources supplied was held fixed in these simulations with parameters as described in Methods. Simulations where the amount of each resource supplied was held fixed instead gave similar results. (PDF) [file pcbi.1010570.s008.pdf]

**A**

bottleneck

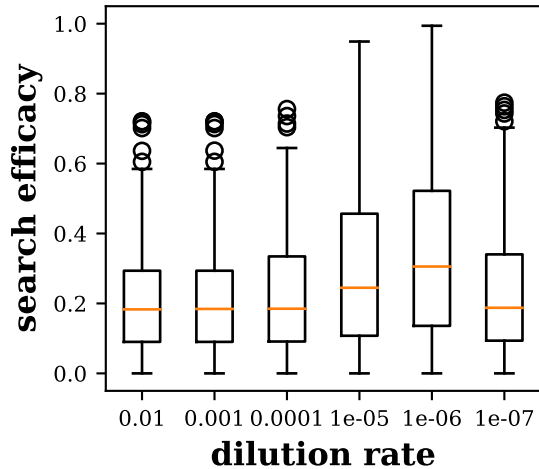**B**

bottleneck+addition

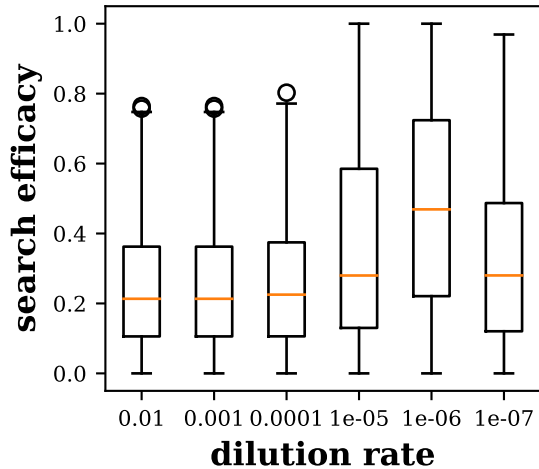

Supplement: S8 Fig — The dilution-based search protocols, ‘bottleneck’ and ‘addition + bottleneck’, have a high search efficacy only if the bottlenecking step kills a few species but not too many. Therefore, it works best for a narrow range of dilution factors where only order 10 cells survive bottlenecking, before being subject to invasion [79]. Community function was the productivity of a pair of species and simulations were the same as in Fig 9. (PDF) [file pcbi.1010570.s009.pdf]

**$\overline{R^2}$  rounded to zero**

1.0  
0.8  
0.6  
0.4  
0.2  
0.0

0.0

0.2

0.4

0.6

0.8

1.0

**$\overline{R^2}$  without rounding**

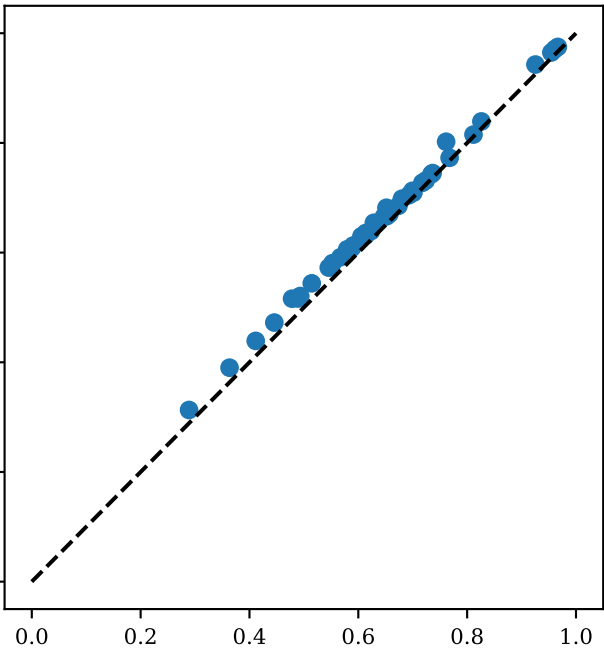

Supplement: S9 Fig — R2¯ after rounding negative abundance predictions to zero is in good agreement withR2¯ computed without rounding negative predictions of the linear model shown in Fig 2. (PDF) [file pcbi.1010570.s010.pdf]

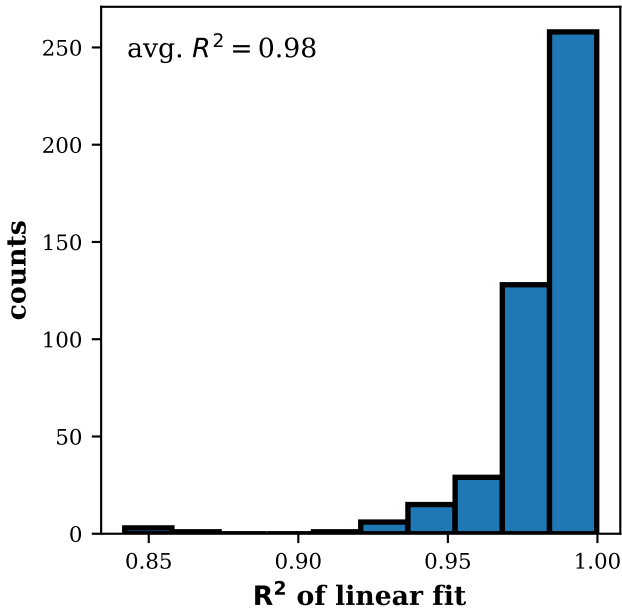

Supplement: S10 Fig — While the experiment by Langenheder et. al. [32] assayed the cumulative metabolic activity of the microbial communities at different timepoints, used the rate of metabolic activity as the community function. The rate of metabolic activity measured as the slope of a linear fit to the cumulative metabolic activity. The rate was at steady state as evidenced by the high R2 of linear fit to the data as shown in the figure. (PDF) [file pcbi.1010570.s011.pdf]

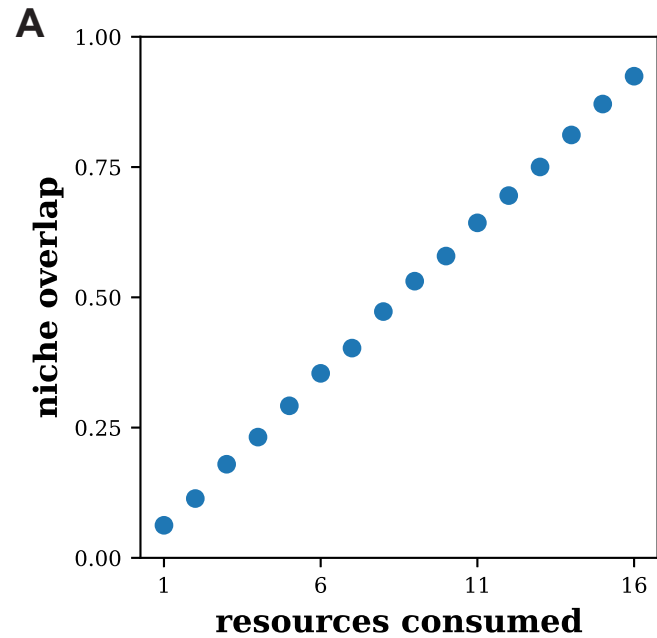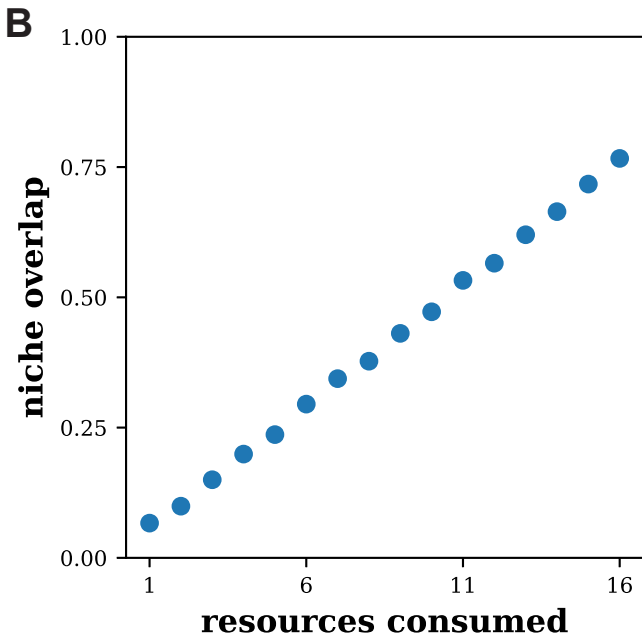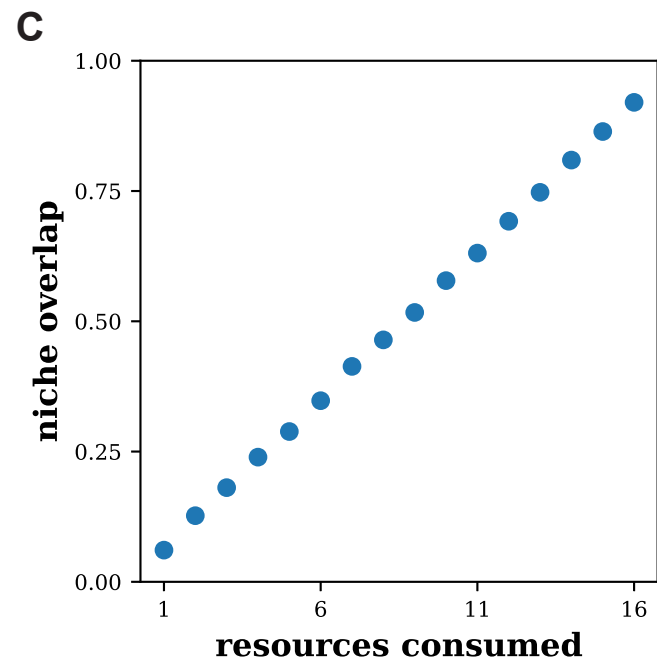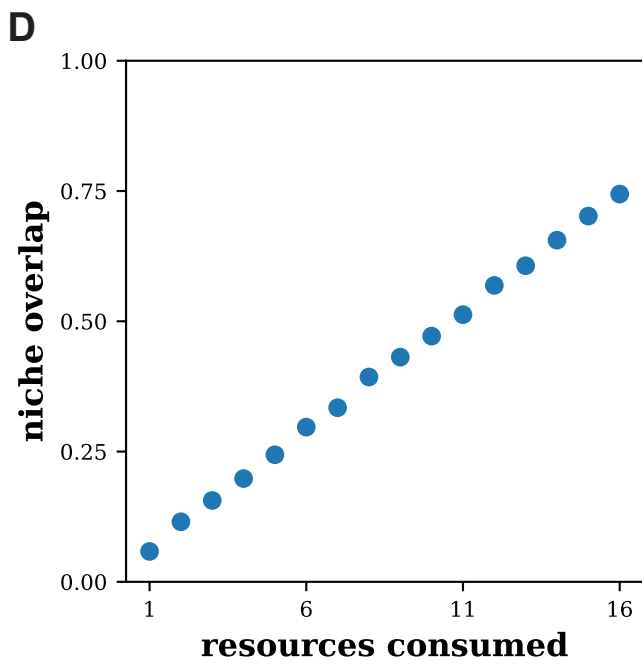

Supplement: S11 Fig — The niche overlap in 16 species pools where the non-zero consumption matrix elements were sampled from a uniform distributions (A,B), and lognormal distributions (C,D). Niche overlap was quantified by the average cosine similarity between the consumption vectors of each species pair in the pool, as in previous studies [33]. The uniform distribution in A extended from 0.5 to 1.5; in B extended from 0.1 to 20.5. The lognormal distribution parameters were μ = 0, σ = 0.3 in panel C and μ = 0.6, σ = 0.6 in panel D. The extent of increase in niche overlap reduces as the variability in the sampling decreases. (PDF) [file pcbi.1010570.s012.pdf]

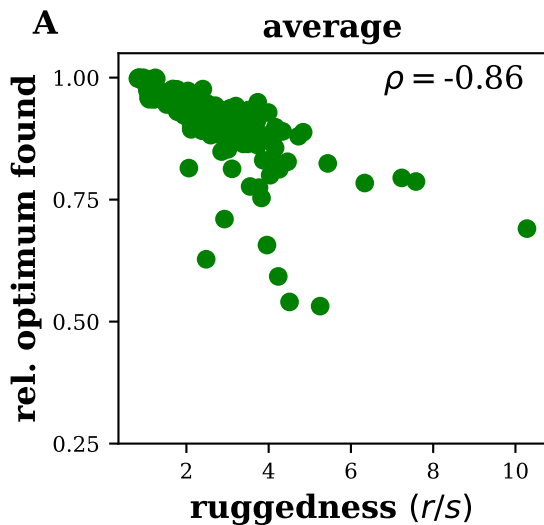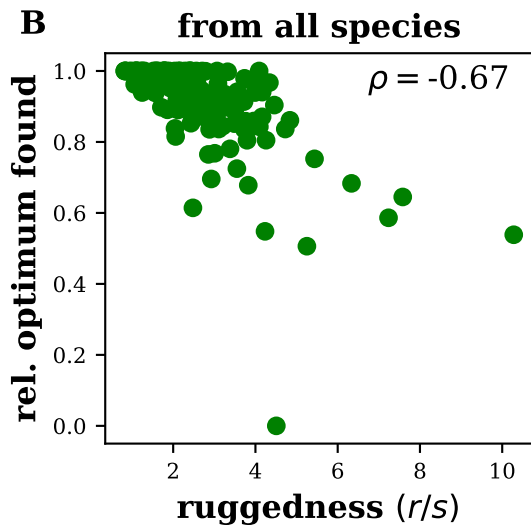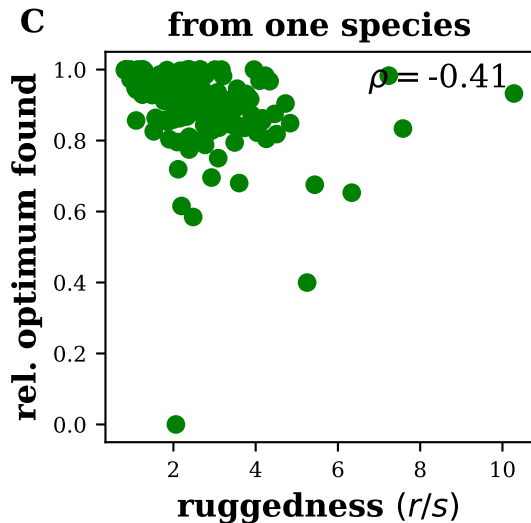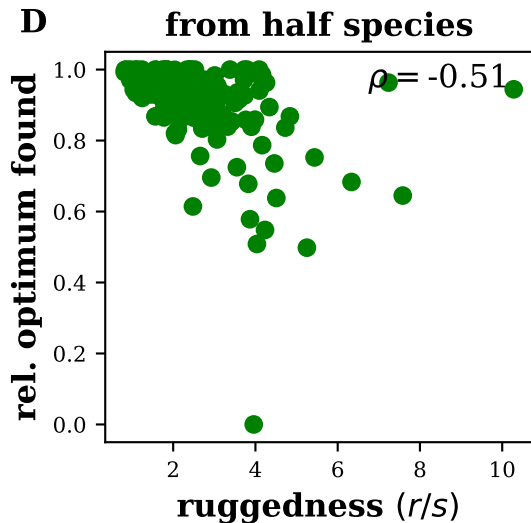

Supplement: S12 Fig — For Shannon diversity, the ruggedness measure is informative not only about the average search outcome (A), but also about search outcome starting from the community with all species present (B), a randomly chosen species in monoculture (C), and from a randomly chosen community with half the candidate species present (D). Simulation data was the same as in Fig 3. (PDF) [file pcbi.1010570.s013.pdf]
