# Supplementary material for: Ecological landscapes guide the assembly of optimal microbial communities
Source: PLoS Comput Biol. 2023 Jan 10;19(1):e1010570. doi: 10.1371/journal.pcbi.1010570 (PMC9831326; doi:10.1371/journal.pcbi.1010570)
Supplement: Attachment — Submitted filename: PLoS Comp Bio review response.pdf [file pcbi.1010570.s014.pdf]

Dear Dr. Bino George,
Thank you very much for submitting your manuscript "Ecological landscapes guide the assembly of optimal microbial communities" for consideration at PLOS Computational Biology.
As with all papers reviewed by the journal, your manuscript was reviewed by members of the editorial board and three independent reviewers. In light of the reviews (below this email), we would like to invite the resubmission of a significantly-revised version that takes into account the reviewers' comments.
In particular, Reviewer 1, while largely positive about the study, raises questions about the effect of niche creation through by-product secretion during community assembly on the results, the measure of resource overlap, and validity of the experimental results (indeed, perfect ant-correlation with 3 points does not permit any reliable conclusions). Reviewer 2 questions the generality of the results, and asks for greater theoretical support for several choices made, including choice the the community function measure. They too question the value of the experimental evidence provided, and the relevance of the theory to it. Reviewer 3, in a similar vein as 2, asks for some robustness analyses, and a more rigorous treatment of the optimization problem underlying maximisation of microbial community functioning. And like Reviewer 1, they too raise the issue of niche creation through by-product secretion during community assembly.

We thank the editor and reviewers for their constructive and detailed feedback. We have made significant revisions to the manuscript in response to the comments. Some of the comments reflect the challenges with which we struggled conducting this type of research. When one wants to figure out some kind of “rules of life”, it is often difficult to select a specific model or experimental system that would be generic enough. Even with a system in hand, the number of conditions, parameters, and hypotheses one could investigate sets one up for an endless exploration. The best we can do is to let the data suggest certain conclusions and then test their robustness. We can then be reasonably confident that the behavior reported is typical even though we do not have a proof that it always holds.

We summarize some major revisions to the manuscript in response to comments highlighted by editors here; detailed responses to individual reviewer comments then follow.

In response to the comments on the choice of community function and robustness of results to other choices of community function, we analyzed an additional community function landscape, motivated by an experimental study of butyrate production by *in vitro* gut bacterial communities [Clark et. al. Nat Comm 2021 doi.org/10.1038/s41467-

021-22938-y]. The study inferred a nonlinear function for butyrate production that depended not only on species abundances but also species presence-absences (lines 536-543), thus setting it apart from the functions considered in the original submission. We used the empirical inferred parameters to generate landscapes from simulated data by randomly assigning species labels. In this way, we hoped to mimic the structural complexity of an empirical landscape. As seen in Figs. 3 and 7, our results remained robust to this novel functional form motivated by experiment. We have also added a number of figures to the SI to demonstrate the robustness of our results as suggested by the reviewers (Figs. S3, S6, S9).

To better quantify niche overlap, we have introduced an additional metric, which is more general than the number of consumed resources. In particular, we have shown that niche overlap and landscape complexity can be controlled by the similarity of the consumption rates and demonstrate the robust increase of niche overlap with number of resources, independent of how these consumption rates are generated in the model (Figs. S1 and S11). Figs. 9, S5, S6, S7 show the effect of varying byproduct secretion, niche overlap, and resources supplied on community properties and demonstrates that ruggedness and search efficacy remain anti-correlated in all scenarios.

We have also made the treatment of the optimization problem more rigorous. We have added a proof of why linear landscapes are optimizable (lines 553-565), a workflow for ruggedness computation (Fig. 4), and a theoretical analysis of the niche-overlap vs linearity relationship in the context of a simplified community model (SI Sec. S5).

In response to the concerns raised about our discussion of the experimental data, we have revised the relevant text. Based on reviewer suggestions, we have also analyzed an additional, incomplete experimental landscape, to report estimated ruggedness of landscapes of short chain fatty acid production measured in the study (Fig.11C) [Clark et. al. Nat Comm 2021 doi.org/10.1038/s41467-021-22938-y].

We cannot make any decision about publication until we have seen the revised manuscript and your point-by-point responses to the reviewers' comments. Your revised manuscript is also likely to be sent to reviewers for further evaluation.
When you are ready to resubmit, please upload the following:
[1] A letter containing a detailed list of your responses to the review comments and a description of the changes you have made in the manuscript. Please note while forming your response, if your article is accepted, you may have the opportunity to make the peer review history publicly available. The record will include editor decision letters (with

reviews) and your responses to reviewer comments. If eligible, we will contact you to opt in or out.
[2] Two versions of the revised manuscript: one with either highlights or tracked changes denoting where the text has been changed; the other a clean version (uploaded as the manuscript file).
Important additional instructions are given below your reviewer comments.
Please prepare and submit your revised manuscript within 60 days. If you anticipate any delay, please let us know the expected resubmission date by replying to this email.
Please note that revised manuscripts received after the 60-day due date may require evaluation and peer review similar to newly submitted manuscripts.
Thank you again for your submission. We hope that our editorial process has been constructive so far, and we welcome your feedback at any time. Please don't hesitate to contact us if you have any questions or comments.
Sincerely,
Samraat Pawar
Guest Editor
PLOS Computational Biology
Kiran Patil
Deputy Editor
PLOS Computational Biology
****

Reviewer's Responses to Questions
Comments to the Authors:
Please note here if the review is uploaded as an attachment.
Reviewer #1: This is an interesting paper that I very much enjoyed reading. I believe it represents an important contribution to the field of microbial ecology. As the authors mention, a number of protocols for the search of optimal consortia have been proposed in the past, but have only been very moderately successful at best. Cementing the ecological landscape framework could be key to develop better strategies, and in that sense this work provides useful tools to understand when and how ecological landscapes can be navigated.

We thank the referee for their comments. We agree with the referee that the ecological landscape framework could be key to develop better search strategies. Indeed, some of us are currently using the landscape framework to develop new search strategies.

I do have some concerns, particularly regarding what the authors refer to as the "density of interactions", the metric they use to quantify it, and the claim of it shaping ecological landscapes. Specific comments follow:

1. In figure 2 and in the entire first section of the Results ("Niche overlap controls the complexity of ecological landscapes") it was not clear to me which model the authors were using (with/without cross-feeding) until the very last sentence of the section. I think it would be helpful to specify this earlier on and in the figure legend. Similarly, I think it would help to describe both models at least minimally in the Model section and not only in the Methods. Specifically, I had to look in the Methods to find that, in the model with cross-feeding, a single resource was supplied externally (versus all resources supplied externally in the model with no cross-feeding). This is completely reasonable, but since I think the difference affects how one should interpret the results and the metrics that are used to describe the ecological properties of the system, I would say it is important to at least mention it in the main text.

We have added a panel to Fig.1 illustrating the consumer-resource model and show model equations in the figure. We now clarify that we use the model without leakage in the caption of Fig. 2 and mention that only a single resource was supplied in the caption of Fig. 9 and associated text (lines 306-310). We also show that a model with leakage with varying number of resources supplied still shows an anticorrelation between search efficacy and ruggedness (SI Fig. S5).

2. The concept of more "overlap in resource utilization profiles" leading to a higher "density of interactions" (lines 144-146) makes intuitive sense to me. However, I think there might be some nuance to it. If I am understanding it correctly, the authors refer to the idea that, if a resource can be consumed by multiple species, there will be competitive interactions among them for said resource and therefore a higher interaction density is expected in the 'generalist' case versus the 'specialist' case (if this is correct, I think explicitly explaining it in the main text would help). However, the role of facilitative interactions (in the model with cross-feeding) in this framework is unclear to me. I could see how, in the model with cross-feeding, a higher degree of specialization could lead to species secreting byproducts that they cannot utilize themselves, thus creating new niches into which additional consumers could be recruited (see e.g. Estrela et al. Diversity begets diversity under microbial niche construction, biorXiv, 2022). Then, one could make the argument that increasing the degree of consumer specialization could increase the density of *facilitative* interactions (and not only decrease the density of *competitive* interactions). It is not obvious to me why one would not expect a high interaction density in the model with cross-feeding with highly specialized consumers, even if these interactions were predominantly facilitative. From this perspective, one could maybe intepret the results in the first section of this paper (and in figure S1) as indicating that "community assembly becomes more predictable as interactions become less competitive" rather than "as the number of interspecific

interactions decreases". In summary, I think that the effect of changing the degree of consumer specialization level could have different effects on the two models in terms of a generic "density of interactions" if one accounts for both competition and facilitation. I might be understanding it wrong, but this was not straightforward for me to understand and I would appreciate if the authors could elaborate on it.

We thank the reviewer for bringing to light this point of confusion. The reviewer is correct that we refer to competitive interactions in the particular section; consumer-resource models without cross-feeding only allow for competitive interactions. To avoid possible confusion, we have revised the use of the term “density of interactions” and added SI Fig. S1 to measure niche overlap.

The density of facilitative interactions is more difficult to control in a simple way. In Fig.9, the density of facilitative interactions varies as the fraction of leaked metabolites increases. However, the relationship between density of facilitative interactions and fraction of leaked metabolites is not straightforward due to the many feedbacks involved. For example, production of any metabolite is a form of facilitation, yet it also creates competition for this metabolite among other species; furthermore, the consumed metabolite could produce further byproducts that again create new cooperative and competitive interactions. Since quantification of all of these interactions is still an open question, we did not attempt to investigate how the density of cooperative interactions affect the search efficacy.

We also think that it is premature to conclude that search becomes more difficult when the communities become more competitive vs. more cooperative. In particular, we have no evidence that highly cooperative communities are easier to optimize than purely competitive communities. Thus, when we describe our results we do not comment on the effect of cooperative interactions but only the effect of varying leakage (Fig.9).

3. I am not sure the ratio between M_consumed and M_total is generally a good proxy for the overlap in resource utilization. If I am correct, the more species that *can* consume a resource, the higher the number of *potential* interactions. But this can depend on, first, how species "choose" to consume resources (if at all), and, second, how much variation in the values of the uptake rates there is (i.e. the width of the distribution from which they are drawn).
In the absence of species-specific resource preference profiles, species essentially consume all resources that they can uptake. But if resource preferences are variable across consumers, species can preferentially utilize a specific resource while it is available, even if they are also able to consume a different one (and, in the more general case, they can exhibit higher preference for a resource that is not necessarily

that for which they have a higher uptake rate). Even assuming no species-specific resource preference profiles, if the variation in the uptake rates is large, it could still be the case that any two species could have similar profiles in terms of the resources they *can* consume (i.e. which uptake rates are non-zero) while still exhibiting substantial differences in the actual values of their uptake rates. In either of these scenarios I could imagine that, once equilibrium is reached, the overlap in resource consumption in the community could be lower than the overlap in which uptake rates are non-zero. In other words, it could happen that species are very similar in terms of the resources they can consume (M_consumed close to M_total) but in practice utilize different resources in the stabilized communities due to differences in their preference profiles or the values of their uptake rates being very unevenly distributed.
I understand that the simulations in this work are carried out under the assumption of no regulation or species-specific resource preference profiles. The implementation of resource preference in consumer-resource models is a problem on its own (e.g. Wang, Goyal et al., Nat Commun 2021) that falls out of the scope of this paper, and I certainly do not think it is necessary for the authors to tackle this issue in their work. I am, however, left wondering if M_consumed/M_total is a good proxy for the overlap in resource utilization profiles in any general scenario. It could be useful to analyze the effect of changing the width and/or skewness of the distribution used to sample uptake rates, or to consider alternative metrics for resource utilization overlap. Alternatively, the authors could further elaborate in the text on why they think M_consumed/M_total is an appropriate metric of niche overlap under their conditions.

This is one example of the numerous choices that need to be made in order to run even a simple consumer-resource model. We have not found any unexpected effects of the distribution or the mean strength of consumption. Therefore, we focused only on the number of the consumed nutrients as a control knob to tune the density of interactions. This is also the quantity that can be most easily accessed experimentally (from genomics or Biolog assays).

Following the reviewer’s suggestions, we now discuss how consumption strength affects the density of interactions. In Fig. S1, we show the results for another metric of niche overlap that explicitly includes the strength of consumption. We use this metric to measure niche overlap when consumption is sampled from other distributions (Fig. S11). In all cases niche overlap increases with number of resources consumed. Hence, due to its transparent definition and relative ease of experimental measurement, we choose to use the number of resources consumed as the measure of niche overlap in the main text.

4. In lines 334-335, the authors write "the search efficacy was perfectly anti-correlated with ruggedness on the three pure carbon sources (Spearman's correlation coefficient rho = -1, p = 0.0)". I'm not sure that a perfect Spearman's correlation of only 3 data points and such small variations in both axis (as the authors themselves point out) is really noteworthy. In fact, even if no correlation was observed, the author's argument would not be invalidated --it could still be the case that other ecological landscapes (perhaps with a different choice of species, environments, and/or functions, as the authors mention) could be more rugged and difficult to navigate. The question that comes to mind looking at figure 10 is how rugged empirical ecological landscapes are. I understand that the authors only looked at the specific dataset from Langenheder et al. because it is a combinatorially complete landscape, but in figure 7 they show that they can obtain good estimates of ruggedness from partial data. Even if they cannot compute search efficacy in a partial landscape, I think it would still be useful to have a sense of how rugged ecological landscapes are in a wider context. Some examples of partial landscapes that I suggest the authors check are:

Clark et al. Design of synthetic human gut microbiome assembly and butyrate production. Nat Commun 12:3254 (2021)

Kehe et al. Massively parallel screening of synthetic microbial communities. PNAS 116(26):12804-12809 (2019)

We thank the reviewer for their comments and suggestions. We certainly did not intend to imply any statistical significance to the correlation of three data points. We have revised the text to eliminate this confusion by deleting that sentence. We have also removed the dashed lines in Fig.11 that highlighted this relationship.

We also find it interesting to see how rugged real ecological landscapes are. This was one of the reasons for including Fig. 11. In fact, Fig. 11b showed that the approximation of a real landscape by models of different complexity exhibits the same nearly exponential improvement in accuracy as our consumer-resource models.

Following the suggestion made by the reviewer, we have analyzed the data from Clark et al and report the ruggedness of their landscapes in Fig. 11C. Since only 500 of the 10^8 possible points are sampled landscape, we cannot run a greedy search on the sampled landscape, as noted by the reviewer. We found that of the five community functions assayed in the study, butyrate production was the least rugged. Interestingly, this is also the community function the authors aimed to optimize in the study.

Reviewer #2: In the submitted manuscript, George and Korolev develop a framework to analyze heuristic strategies used to determine the composition of microbial communities that optimize a given ecological function.
While the content of the manuscript is mostly scientifically valid (with some caveats, see points below), the results are not of high importance to the researchers in the field. In fact, the results found by the authors rely on several arbitrary choices (e.g., which ecological function to optimize) and it is not obvious how their conclusions would generalize to different choices. Therefore, publication in PLoS Computational Biology cannot be recommended.

If we understand correctly, the reviewer’s main concern is that our results may not generalize. Because, if they do, then clearly many researchers in academia and industry would benefit from using landscape framework and other results to optimize their microbial communities more effectively.

As far as generality, assumptions and choices are part of any biology research. Strictly speaking, any experiment with E. coli can only tell us about the structure and function of the very specific E. coli strain used in the experiment. Yet, there is great success in generalizing such observations to other bacteria. And, yes, sometimes what we find for E. coli does not hold for other organisms and this disagreement leads to further experiments and deepens our understanding of biology.

Just like shared ancestry allows us to apply results from E. coli to other microbes, the prevalence of competition for consumable resources suggests that consumer-resource models could be informative for many ecosystems. Within the framework of consumer-resource models, we tested the robustness of our results to various assumptions. The revised manuscript also includes further tests suggested by the reviewer.

We have also analyzed a new ecological function, motivated by the inferred functional form for butyrate production by *in vitro* gut microbial communities. The dependence of this function on species presence-absences as well steady-state abundances differentiates it from the other community functions. We observed all our main results to hold robustly for this choice of function as well.

The manuscript would, however, be suitable for publication in other journals like PLoS ONE, provided that the points covered below are appropriately addressed:

1) Some of the references cited by the authors in Table 1 (e.g., [27] and [28]) do not support their claims. The authors should choose more carefully which references to use in that Table.

We thank the reviewer for this comment. This and other comments have led us to revise the table significantly. Additionally, we have added SI Sec. S1 which summarizes the results of each group of references. At the same time, we believe the references mentioned by the reviewer are appropriate for the table.

Ref. 27 (now Ref. 33) is one of the original MacArthur papers on the consumer-resource model. In this paper, MacArthur showed that community assembly in consumer-resource models always increase an objective function, resembling a Lyapunov function in dynamical systems. The existence of such a function implies that the community converges to a single steady state that depends only on species presence-absences. Refs. 34 and 35 discuss this point in greater detail.

Reference 28 (now Ref. 50) identifies conditions under which a community will converge to a single steady state, and conditions under which more complicated dynamics (such as oscillations and chaos) are exhibited. For this reason, we had cited this paper in two rows of the table: “single steady-state” and “complicated dynamics”. To prevent further confusion, we now cite this paper only in the row pertaining to complicated dynamics.

We hope that the revised table and Sec. S1 will help clarify some of these points of confusion.

2) In lines 184-185, the authors state “The precise definition of the diversity did not affect our conclusions, so we focused on the Shannon Diversity”. This statement is not substantiated by any result. The authors should provide concrete examples that their statement is indeed true (i.e., show that their results do not change with different definitions of diversity).

We did not show these figures for brevity. We did carry out the necessary simulations though and found that the results hold for different standard metrics of diversity. We have rephrased this sentence and mention the correlation of ruggedness with another measure of diversity, Simpson’s Diversity, in the text (lines 527-529).

3) In several parts of the manuscript (e.g., Fig. 3, Fig. 6) the authors show results relative to the ecological functions “pair productivity” and “focal abundance”. Contrarily

to “diversity” (the other one they consider), these two functions depend by definition on the choice of one or two particular species in the community. The authors, however, never specify if the results they are showing are relative to a particular choice of species or if they are an average over different choices. This fact should be stated clearly whenever they show results relative to “pair productivity” and “focal abundance”.

The community function of pair productivity and focal abundance were defined for a particular choice of random species. We had mentioned this in the initial manuscript version in lines 184-188, the Methods section, and Eq. (8). We have now added an additional sentence in the Methods to further clarify this (line 531).

Also, it is important to note that in each pool, species are parameterized randomly. Therefore, species with the same index in different pools are not correlated in any special way. The results report the average over the different species pools at each set of simulation parameters.

4) In Fig. 4 and lines 229-231, the authors claim that all metrics of ruggedeness increase with niche overlap. However, this is clearly not true for Fig. 4B since the metric quickly saturates to a constant value. This discrepancy should be addressed more carefully in the manuscript. Furthermore, the authors show in Fig. S2 the equivalent of Fig. 4 for another ecological function (pair productivity) but never show the same results for focal abundance. This result should be included in the manuscript to substantiate the author’s claims, and all the plots should be put together in the Main Text instead of being separated between Main Text and Supplementary Information.

The difference in the increase of ruggedness with niche overlap is because the various ruggedness metrics quantify different aspects of landscape structure. Line 232 in the initial manuscript mentioned this. We have now added text to clarify this further (lines 253-257).

We have added SI Fig. S3 showing the same overall trend holds for all community functions. We choose to keep these extra figures in the SI for clarity and brevity.

5) The plot shown in Fig. 5 is not really a surprising result, since it's showing that increasing the order of the function used to fit a model increases the accuracy of the results. This plot is a nice control of the validity of the author's results, and should be moved to the Supplementary Information instead.

Fig.5 is making two additional points that aren't trivial: 1) the decrease in unexplained variance seems to be approximately exponential for these ecological models. 2) it demonstrates the difference in this curve between specialists and generalists. Furthermore, we saw a similar, approximately exponential decay in the experimental landscape. While we do not yet understand why, this decay may indicate some general structure underlying ecological landscapes.

6) In the paragraph starting at line 271, the authors claim that their results generalize to other models and search protocols. While what they show substantiate this claim for different search protocols, the authors are not really considering different models: they are simply keeping the same model they've been using in the rest of the manuscript (i.e., the consumer-resource model) and modified with the addition of cross-feeding. However, this is still a consumer-resource model and not a different one. The authors should change the wording of that paragraph and state that the results generalize to consumer-resource models with cross-feeding (and not "to other models" in general).

We changed the title to "Results generalize to other search protocols and the presence of cross-feeding".

7) In Fig. 8B, the search efficacy exhibit a very large variation for a fixed value of the metabolic leakage l (e.g., the distribution shown for l=0.2 goes from 25% to 100%). This fact should be discussed in detail by the authors, since such a large variation could make the heuristic search strategy worthless. Furthermore, the results shown in this figure are relative to only one of the ecological functions that the authors consider in the manuscript (i.e., the focal abundance). The authors do not show anywhere in the manuscript (including the Supplementary Information) the results for the other two ecological functions they consider. In order to substantiate their claims, these results should be shown alongside Fig. 8.
The search efficacy varies due to both the particular realization of the species pool and the set of simulation parameters. This random variation can create some hard to optimize landscapes, like the particular landscape l=0.2. However, this does not mean that there is a systematic trend of the search efficacy with the parameter. Notably, as Fig. 8D shows, this landscape was very rugged and so had a low search efficacy.

We have added an SI figure for the other community functions (SI Fig S6).

8) The application of the model to experimental data that the authors show in the paragraph starting at line 315 is very weak: by the author's own admission, the results are not statistically significant. This part of the manuscript should either be removed or supported with a more carefully executed experiment. For example, the dataset they use contains only six species, while all the results that the author showed about the efficacy of the heuristic search strategies are relative to communities with 16 species. Since the dimensionality of the system's landscape changes dramatically if we have 6 or 16 species, the authors should discuss in detail how a different species pool size affect the application of their model to experimental data.
Furthermore, in lines 331-333 the authors state "Because of the rather simple metabolic interactions in these communities, the community landscapes were quite smooth and the search efficacy was quite high". This claim, however, is not substantiated by any result.

The data is from a previously published dataset. This was the largest complete landscape we could find in experimental data. We have now also analyzed the partial landscape from [Clark et. al. Nat Comm 2021 doi.org/10.1038/s41467-021-22938-y], made by 25 species, and added panel C to Fig.11 as well as text (lines 363-373)

We have removed any mention of statistical significance of the anti-correlation between the three experimental points. We have also revised the discussion of the analysis of experimental data (lines 358-362 and caption of Fig.11).

9) In the paragraph starting at line 402, the authors show how they have chosen the parameters used in the simulations. Several of those parameters have been drawn from particular distributions (e.g., gamma distributions). While this choice per se is not problematic, the authors should explain why they have made this choice, and show what happens if the parameters are drawn from other distributions (e.g., a normal, log-normal or uniform distribution).

We made the choice of a gamma distribution motivated by previous work [Marsland R, et al. (2019) PLoS Comput Biol 15(2): e1006793], and now mention this (line 474).
We have now quantified niche overlap using an alternative measure in SI Fig. S1. We use this measure to show how niche overlap can varies with different distributions, such as uniform and lognormal (Fig. S11). In all the considered cases, niche overlap increased with the number of resources consumed.

10) In line 450, the authors state “This truncation did not affect our results” but do not show any result that substantiates this claim.

We have added Fig.S9 to show that our results are unaffected by truncation.

Reviewer #3: George and Korolev studies the effectiveness of heuristic gradient-ascent type approaches to search species in microbial communities in order to optimize community functions. Using simulations from consumer resource models, they demonstrate that (1) search effectiveness is inversely related to the ruggedness of the underlying ecological landscape, (2) communities with increased niche overlap tend to be more rugged, and (3) ruggedness can be estimated with limited and noisy data. I think the current manuscript is difficult to follow without prior exposure to the concept of landscape ruggedness and I suggested several ways to improve clarity and to make connection with established concepts in optimization theory (Major comments 1, 5, 6, 8). There are also additional robustness analysis that I hope the authors could perform to improve relevance to practice (Major comments 2, 3).

We thank the referee for their detailed comments and summary of our work.

Major comments:
1. The clarity of this manuscript could be enhanced by including a step-by-step flow chart of how ruggedness is computed. The current description of ruggedness is either too vague or too detailed.

We thank the referee for this suggestion. We have now added a flow chart illustrating how ruggedness is computed (Fig. 4).

2. I would encourage the authors to perform some additional robustness analysis of the results in Figure 7. This includes, for example, considering models that include non-resource mediated interactions, such as pH-mediated interactions. These interactions are described by non-monotone functions hence may challenge the ability to predict ruggedness (or roughly speaking convexity of optimization problem) from limited data.
We address comment 2 along with comment 3 below.

3. Related to my previous comment, the consumer resource model used in this paper does not account for sequential resource uptake or growth regulation by multiple metabolites/resources. These growth models are common for bacterial growth and considering them could demonstrate the robustness of the results and significantly improve the relevance of these computational results to experiments.

We thank the referee for these comments. We agree with the referee that the consumer-resource models studied in the manuscript do not account for non-resource mediated interactions, such as pH, and sequential resource uptake. These are certainly great suggestions for future work. We now discuss these limitations of our work in both introduction (lines 68-78) and in the discussion (lines 408-420).

In this manuscript, we focus on consumer-resource models because they are well-characterized. Hence, we can generate reasonable parameters and explore their effects on the search dynamics. pH-based interactions are certainly interesting and not captured by consumer-resource models. However, we are not aware of a well-accepted model of pH-based interactions in microbial communities that could be used to carry out our analysis. The development and analysis of a general model of pH-based interactions would amount to a new study.

In this context, we would also like to highlight the recent paper on *in vitro* gut bacterial communities [P.Y. Ho, T.H. Nguyen, et. al. Biorxiv 2022 doi.org/10.1101/2022.05.30.494065]. The work shows that resource competition explains majority of the interactions between gut bacterial species. Consumer-resource models have also been able to reproduce many statistical features of communities [Marsland, Cui, Metha Sci Rep 2020; Ho, Good, Huang eLife 2022].

Sequential resource update or diauxie is important as well but is not included in consumer-resource models. Although recent work (in collaboration with author A.B.G.) has made progress towards developing a consumer-resource model of diauxic species [Z. Wang et al. Nature Communications 2021 doi.org/10.1038/s41467-021-27023-y], the model requires further exploration before being widely accepted. Further, the metabolic constraints and resource supply conditions that cause sequential utilization of resources (c.f. [H. Okano, R. Hermsen and T. Hwa 2021 doi.org/10.1016/j.mib.2021.07.008]) needs to be better understood to parameterize such a model appropriately.

Importantly, sequential nutrient uptake may not have a significant impact on the chemostat dynamics simulated here. In a chemostat, the system reaches a steady state, where resource abundances attain a constant value and supplied resources are never fully depleted. Therefore, microbes will not shift the resources they consume. Hence resource preferences mostly affect the initial transient dynamics towards the steady state. The steady state is then characterized by the “realized” consumption rates, which is quite similar to the assumptions of the consumer-resource models.

We were a little confused by the term ‘growth regulation by multiple resources’. In our model growth of generalist species depend on multiple resources and uptake rates take

the Monod form in simulations of the cross-feeding model. If ‘growth regulation by multiple resources’ instead meant growth of the form of Liebig’s law of the minimum, we did not consider this scenario because it can lead to strong violation of our assumptions. As we mention in the table, the classic works by Huismann and Weissing [doi.org/10.1890/0012-9658(2001)082[2682:BCFOAC]2.0.CO;2] show that consumption of more than 3 essential resources can lead to complex dynamics such as heteroclinic cycles and chaos, which is not considered within the ecological landscape framework.

4. The summary in Table 1 lacks mathematical rigor and needs to be seriously re-considered. For example, multi-dimensional Lotka-Volterra models always have multiple steady states, as opposed to “single steady state” shown in the table. While there is only one positive equilibrium, the other equilibria with at least one species being 0 (i.e., extinct) could still be stable. “Typically a single steady state” needs to be clarified. Does it mean the number of steady states depends on parameter? Additionally, what is relevant to practice is the number of stable steady state instead of the number of steady states. This needs to be clarified in the table. In general, I think the contents in Table 1 are over-generalizing the complicated behaviors that could arise from nonlinear dynamical systems and needs to be made precise mathematically if the authors want to keep them in the manuscript.

We thank the referee for bringing to our attention the many points of confusion with Table 1. We have now substantially revised the Table and references to prevent confusion. We have also added an entire section to the SI (Sec. S1) that discusses the content of Table 1 in greater detail.

We clarify that we refer only to stable steady states since only these are relevant for our argument. One can obtain additional steady states by removing a species; however, if those states are unstable to the introduction of the species that was removed, then we consider them unstable. Thus by ‘stable steady-state’ we mean that the steady-state obtained as outcome of the dynamics is stable with respect to the reintroduction of any species that was present in the starting community. We include this explanation in the caption of the Table.

The Lotka-Volterra models we now refer to have either a Lyapunov function or fall in the class of randomly parameterized Lotka-Volterra models studied using spin glass techniques by the works of G. Bunin, G. Biroli, D. Kessler, N. Shnerb and others. The studies of these randomly parameterized Lotka-Volterra models have found phase diagrams, such as Fig.1 in [F. Roy et al J. Phys A 2019 doi.org/10.1088/1751-8121/ab1f32], which classify model behavior. Notably, they find that in regions where the scaled variance of species interactions is small dynamics converge to a unique

equilibrium. When interaction variance crosses a threshold value the system exhibits multiple attractors with potentially unstable directions. The stochastic immigration events lead to complex dynamics that resembles chaos in this parameter region.

We have clarified what we mean by “typical” in the Table. In the references cited in that category, only a few percent of the initial conditions resulted in multiple steady states.

We explicitly state that the table only approximately condenses the vast literature in the field and direct the interested reader to the cited references for a more rigorous understanding.

5. While the inverse relationship between ruggedness and search efficacy is clear through numerical simulations, the quality of this paper could be significantly improved if the authors could provide some analytical insights to this phenomenon, especially by making connections to concepts in optimization.
If I understand correctly, in its very basic form, most of the ruggedness measures of F quantify how close the function F could be approximated by a linear function of the absence/presence of species (i.e., vector \sigma). One of the main results in this paper is that the objective function evaluated at the steady state of the consumer resource model with small ruggedness (hence close to a linear function) tend to be easier to optimize using a heuristic search that resembles gradient-ascent. This result could be investigated/discussed in the context of optimization theory. In particular, consider the ideal case where the objective function is linear, is there any theory/heuristic from integer programing literature that suggests convergence of the gradient-ascent algorithm to global maximum? More generally, how ruggedness is connected to the convexity of an optimization problem, which I think is a more well-defined/well-known mathematical property compared to ruggedness? Perhaps such connections have been made clear already in the literature and the authors are encouraged to discuss/describe them in this manuscript. This will significantly help understanding and diminish ambiguity.

We thank the referee for these comments. We now demonstrate that a linear landscape is optimizable by a greedy search (lines 553-565). In brief, the optimizability of the linear landscape arise from its separability, i.e., each component can be optimized separately.

The notion of convexity is usually applied to optimization of functions defined on a continuous domain. In the case of functions on a discrete domain, such as our binary vectors sigma, convexity is often used to describe functions that have a convex, continuous analog [Murota, K. (2009). Recent Developments in Discrete Convex

Analysis. doi.org/10.1007/978-3-540-76796-1_11]. The linear objective function is such an example. However, convexity is not necessary for a function to be optimizable by greedy search on a discrete domain.

There is some abstract theoretical research on defining the class of functions optimizable by greedy search on discrete domains, which we now cite in lines 563-565, such as [Helman et. al. An Exact Characterization of Greedy Structures.10.1137/0406021]. But this research is not accessible enough to discuss extensively in our work. The problem of identifying landscape features that quantify optimization difficulty is hard in general and investigations, such as ours, of each class of optimization problem are required to identify useful measures [K. Smith-Miles, and L. Lopes doi:10.1016/j.cor.2011.07.006].

6. The inverse relationship between resource overlap and the ability of the linear model (1) to predict community assembly outcome is interesting. Is it possibly to develop some physical intuition of this using analytical solutions to the consumer resource model for a simple two-species community with and without niche overlap?

We thank the referee for this suggestion. For two species, the linear model will always provide a perfect fit because the number of parameters predicting the abundance of a species in the linear model (2) equals the number of initial conditions in which the species can be present (2). Hence, we analyzed the limiting scenarios of the community model instead to provide some intuition.

Since analysis of the consumer-resource model becomes difficult analytically in all but the simplest scenario, we also analyzed the generalized Lotka-Volterra model (Sec. S5). In this section, we demonstrate why increasing niche overlap makes it harder for the linear model to predict community assembly.

In short, we analyzed the model in the regime that permits stable steady-state species coexistence for all the possible initial species combinations. The steady state of the model for each initial condition is found by inverting a different sub-matrix of the full community interaction matrix. The relation between the different steady states are thus captured by the inverses of the different sub-matrices. We show that this relationship can be fit by the linear model when niche overlap is zero. When niche overlap is high, the relationship between the steady-states become more complex and harder to fit with a linear model.

7. The authors may want to discuss alternative search methods that could be more effective in a rugged landscape.

We thank the referee for suggesting this important discussion point. Effective search strategies on rugged landscapes is a major challenge in modern computational research, which is actively studied in many disciplines. While there are some algorithms that provide advantage in certain settings, we are not aware of an algorithm that would work universally---particularly in the context of ecological landscapes where the number of communities that one can study is much smaller than the number of steps that a computational algorithm can make.

Developing new search strategies tailored to the constraints of ecological searches is certainly an extremely interesting direction, however. Indeed, one of the authors is currently involved in developing better methods for rugged ecological landscapes. We have now added a substantial discussion on this point in lines 425-441. However, a full exploration of these alternatives will be presented elsewhere once that research is completed.

8. The clarity of this manuscript could be benefited substantially by moving some key mathematical contents, such as the consumer resource model and the definition of ruggedness in the Methods section to the main text. The descriptions of ruggedness by words in the main text is very difficult to follow. In addition, I strongly encourage the authors to include a precise mathematical formulation of the optimization problem at hand (e.g., F is the objective function, \sigma vector, taking values in {0,1}^S is to be optimized, and subject to the consumer resource dynamics). The current description of this core optimization problem in line 112-125 makes it difficult to link physical properties to mathematical notations.

We thank the referee for this detailed comment.
We have added a panel to Fig.1 to describe the consumer-resource model better and provide model equations in the figure.
We have added a figure to illustrate the workflow for computing ruggedness (Fig. 4) and provide the mathematical definition of the ruggedness measure *r/s* in this figure.
We have also revised the description of the optimization problem as suggested (lines 131-132).

Minor comments:

1. Is niche overlap discussed in line 149-153 affected by the magnitude of parameters in the consumption matrix?

Niche overlap always increases with the number of resources consumed. We have now added SI Figs. S1, S11 to show the dependence of niche overlap on the number of resources consumed when the consumption matrix is parameterized by different distributions and parameter ranges (and hence different magnitude of elements). In all cases, the niche overlap increases, at different rates, with the number of resources consumed.

2. The model in equation (1) should not contain the "model error" part (\epsilon_i). This is only needed if the model itself is stochastic. However, here the model is deterministic and fitting residue should not be considered part of the model.

We thank the referee for pointing this out. We have now changed this.

3. The connection between N in line 136 and the general function F is unclear. My understanding is that N is an instantiation of F. If this is correct, please state it explicitly.

N is a vector while F is a scalar. In general, one cannot optimize over vectors, so the only F can be used as the optimization quantity. One can, however, view individual components of N as an instance of F. This is what we denote by focal species abundance. We have clarified this relationship between N and F in Methods lines 532-534.

4. Line 157-158: Please indicate what data are used for fitting (i.e., steady state abundance generated from simulation consumer resource models, if I understand correctly).

We have added this (lines 165-166)

5. Line 166: I do not understand what "community assembly becomes simpler" means. Does it mean that steady state community composition becomes easier to predict using a linear model of species absence/presence?

Yes.

6. What does the color bar in Figure 2A-B (abundance predicted vs. observed panels) represent?

It indicates the density of points, estimated by gaussian kernel density estimation. We have added this explanation to the caption.

7. In line 109-111, the statement that “multistability are more of an exception” in microbial communities is rather controversial and may need to be toned down. The authors are also encouraged to discuss how the current method fails in the presence of multi-stability.
We have revised this sentence.

8. What does the color shades in Figure 6D mean?
Color indicates magnitude of correlation. We have added this to the caption.

9. Fig. 10A and Fig. 10B could switch position since 10B is referred first in the text.
We prefer the current order for clarity.

10. The definition of \bar{F} is unclear in equations (11) and (13). What is the mean function taking average over? Is it averaging the objective function F evaluated at all possible values of \sigma?
Yes, \bar{F} is the average of F over the landscape. We have now clarified this in the text (lines 547-549).

Have the authors made all data and (if applicable) computational code underlying the findings in their manuscript fully available?
The PLOS Data policy requires authors to make all data and code underlying the findings described in their manuscript fully available without restriction, with rare exception (please refer to the Data Availability Statement in the manuscript PDF file). The data and code should be provided as part of the manuscript or its supporting information, or deposited to a public repository. For example, in addition to summary statistics, the data points behind means, medians and variance measures should be available. If there are restrictions on publicly sharing data or code —e.g. participant privacy or use of data from a third party—those must be specified.
Reviewer #1: No: Extracted from the review file: "The authors will share the code used to generate and analyze the data in the manuscript upon manuscript publication"
Reviewer #2: No: The code was not available with the manuscript
Reviewer #3: No: I did not find sufficient information about sharing of code and data provided in the manuscript
PLOS authors have the option to publish the peer review history of their article (what does this mean?). If published, this will include your full peer review and any attached files.

If you choose “no”, your identity will remain anonymous but your review may still be made public.

Do you want your identity to be public for this peer review? For information about this choice, including consent withdrawal, please see our Privacy Policy.
Reviewer #1: Yes: Juan Diaz-Colunga
Reviewer #2: No
Reviewer #3: No
